# Immunohistochemical expression of epithelial and stromal immunomodulatory signalling molecules is a prognostic indicator in breast cancer

**DOI:** 10.1186/1756-0500-5-110

**Published:** 2012-02-21

**Authors:** Elin Richardsen, Rebecca Dale Uglehus, Stein Harald Johnsen, Lill-Tove Busund

**Affiliations:** 1 Department of Clinical Pathology, University Hospital of Northern Norway, N-9038 Tromsø, Norway; 2 Department of Medical Biology, University of Tromsø, N-9037 Tromsø, Norway; 3 Department of Neurology and Neurophysiology, University Hospital of Northern Norway, N-9038 Tromsø, Norway; 4 Department of Clinical Medicine, University of Tromsø, N-9037 Tromsø, Norway

## Abstract

**Background:**

The immune system has paradoxical roles during cancer development and the prognostic significance of immune modulating factors is controversial. The aim of this study was to determine the expression of cyclooxygenase 2 (COX-2), transforming growth factor-beta (TGF- beta), interleukin-10 (IL-10) and their prognostic significance in breast cancers. Ki67 was included as a measure of growth fraction of tumor cells.

**Methods:**

On immunohistochemical stained slides from 38 breast cancer patients, we performed digital video analysis of tumor cell areas and adjacent tumor stromal areas from the primary tumors and their corresponding lymph node metastases. COX-2 was recorded as graded staining intensity.

**Results:**

The expression of TGF-beta, IL-10 and Ki67 were recorded in tumor cell areas and adjacent tumor stromal areas. In both primary tumors and metastases, the expression of COX-2 was higher in the tumor stromal areas than in the tumor cell areas (both *P *< 0.001). High stromal staining intensity in the primary tumors was associated with a 3.9 (95% CI 1.1-14.2) times higher risk of death compared to the low staining group (*P *= 0.036). The expression of TGF-beta was highest in the tumor cell areas of both primary tumors and metastases (both *P *< 0.001). High stromal expression of TGF-beta was associated with increased mortality. For IL-10, the stromal expression was highest in the primary tumors (*P *< 0.001), whereas in the metastases the expression was highest in tumor cell areas (*P *< 0.001). High IL-10 expression in tumor- and stromal cell areas of primary tumors predicted mortality. Ki67 was higher expressed in tumor stromal areas of the metastases, and in tumor cell areas of the primary tumors (*P *< 0.001). Ki67 expression in tumor cell areas and stromal areas of the metastases was independently associated with breast cancer mortality.

**Conclusions:**

Stromal expression of COX-2, TGF-beta and Ki67 may facilitate tumor progression in breast cancer.

## Background

Epithelial-stromal interactions are important for tumor development and progression [1]. The stroma surrounding solid tumors contains activated and recruited cells like fibroblasts, innate and adaptive immune cells, and endothelial cells which can be supportive and responsive agents in tumorigenesis [1,2]. An abnormal stroma may cause dysfunction of epithelial-mesenchymal interactions which promotes progression of preneoplastic lesions to malignancy [3]. Changes in the stroma environment may lead to selection of cells with altered survival characteristics. In normal mammary tissue stroma plays a major role in control and regulation of physiological processes in the breast [4]. The complexity of stromal reaction and the signalling mechanisms between tumor and stromal cells in breast cancer is incompletely understood, not least because stroma is continuously remodelled during tumor progression [5,6].

The cytokine cyclooxygenase-2 (COX-2) is frequently expressed by cancerous cells. It is Not constitutively expressed, but can be rapidly induced by oncogenes, other cytokines, chemokines, growth factors, hypoxia, ultraviolet light, and epidermal growth factors. The transcription factors, nuclear factor-κB (NF-κB), hypoxia-inducible factor 1α (HIF1α), and activator of transcription 3 (STAT3) coordinate the production of COX-2 and prostaglandins [7-11]. In early tumor outgrowth elevated transforming growth factor-β (TGF-β) is tumor suppressive, whereas at later stages it may act as a promoter of tumor progression [12,13]. TGF-β induces α-smooth muscle actin and collagen production in culture fibroblasts [14] and is a potential mediator of desmoplastic responses in tumors. Desmoplasia in invasive tumors and metastases is morphologically characterized by extensive proliferation of fibroblast-like cells and extracellular matrix (ECM); inflammation and immune responses represented by lymphocytes, macrophages, dendritic cells; and tumor angiogenesis [15]. Loss of TGF-β sensitivity in carcinoma cells is frequently accompanied by increased expression of TGF-β in the same cells [16]. TGF-β is elevated in cancer cells compared to normal epithelial cells, and appears to be even more elevated in poorly differentiated tumors [17,18].

The significance of interleukin 10 (IL-10) within the tumor microenvironment is debated because it is dependent of the malignant cells, tumor-infiltrating macrophages and lymphocytes [19,20]. IL-10 derived from regulatory T-cells in the tumor or from cells in the tumor microenvironment may stimulate tumor progression [21,22]. In tumor tissue, IL-10 has both immunosuppressive properties (potentially cancer promoting due to inhibitory effects on antigen presenting capacity) and anti-angiogenic properties (potentially cancer inhibiting) [23-25].

Ki67 is a nuclear protein, which is expressed during all the active phases of cell cycle (G1, S, G2 and mitoses), but is absent from the resting phase (G0). Ki67 is strictly associated with cell proliferation during the cell cycle interphase. The Ki67 antigen can be exclusively detected within the cell nucleus, whereas in mitosis most of the protein is relocated to the surface of chromosomes. Studies have shown that Ki67 is associated with tumor aggressiveness in breast cancer [26].

Previous studies have highlighted the epithelial-stromal interactions and emphasized the stromal-epithelial interface as critical mediators of tumor progression [27-31]. In this study, we evaluated the prognostic significance of the immunomodulatory signalling molecules COX-2, TGF-β, IL-10 and Ki67 in tumor epithelium and stromal areas of human breast cancer.

## Methods

### Patients

Primary tumor tissues and tissues from the corresponding lymph node metastases were investigated in 38 untreated breast cancer patients. The specimens were diagnosed at the University Hospital of Northern Norway (UNN) from 2000 to 2003. The initial diagnoses were based on fine-needles biopsies, lumpectomy specimens and resection specimens. To be included, patients must have a confirmed diagnosis of breast cancer. In case of death, the Causes of Death Registry/Population Registry of Norway was consulted regarding the assumed cause of death. Follow-up time was assigned from the date of initial diagnosis to the beginning of February 2010 with date of death as censoring points. The Regional Committee for Research Ethics approved the study. The Regional Committee approved that written consent from the patients for their information to be stored in the hospital database and used for research was not needed because most of the material was more than ten years old and many of the patients are now dead.

### Tissue samples

Representative formalin-fixed paraffin-embedded tissue blocks were obtained from the archives of the Department of Pathology at UNN. Clinical, biochemical and radiological observations up to the diagnosis of metastases were from the patients journals. Histological classification and grading of breast cancer was made in accordance to the World Health Organizations criteria and TMN-classification of malignant tumors [32,33]. Histological diagnoses were ductal carcinoma (34 cases) and lobular carcinoma (4 cases). Hormone receptor status was recorded at the time of the initial diagnose. Histological verification of metastases to axillary lymph nodes was performed at the Department of Pathology, UNN.. The material was collected from our approved biobank for paraffin embedded material and slides. All material was anonymously collected. The data were analyzed anonymously.

### Immunohistochemistry

Antigen retrieval of COX-2 and IL-10 was performed with Protease I, in a final dilution of 1:100 for 4 and 16 minutes, respectively. Antigen retrieval of TGF-β was performed in a microwave oven with Tris/EDTA buffer, pH 9.0, for intervals of 2 × 10 min. Antigen retrieval of Ki67 was performed with steamer with Citrate buffer, pH 7, for 32 min. The slides were then transferred to a Ventana Benchmark^®^, XT automated slide stainer (Ventana Medical System, France). Tissue sections were incubated with primary polyclonal goat antibody against COX-2 (final dilution 1:100), monoclonal rabbit antibodies against TGF-β (final dilution 1:50) and monoclonal mouse antibodies against IL-10 (final dilution 1:20). All antibodies were from Santa Cruz Biotechnology Inc, CA, USA. The mouse monoclonal antibody against Ki67 was optimized for use in a Ventana automated slide stainer in combination with Ventana detection kit. As secondary antibodies, biotinylated goat-anti-mouse IgG and IgM, and goat-anti-rabbit IgG, both 200 μg/ml, were used. For endogenous peroxidase blocking, the I-VIEW™ DAB Detection Kit (Ventana) was used. Finally, all slides were counterstained with haematoxylin to visualize the nuclei. For each antibody, including negative staining controls, all staining was performed in a single experiment. As negative staining controls, the primary antibodies were replaced with the primary antibody diluents. Appropriate positive and negative controls were included in each antibody run according to the manufacturer's recommendations. Single stromal cells within groups of epithelial cancer cells were defined as belonging to tumor cell areas. The tumor stromal areas were defined as stromal tissue surrounding groups of epithelial cancer cells in central parts of the tumor and were negative by IHC staining with antibodies directed against Cytokeratin (CK, Ventana). Stromal tissues in the periphery of the tumors were not investigated. For stromal cell characterisation, the slides were stained by Masson Trichrome (collagen fibres), Giemsa (granulocytes), Vimentin (fibroblasts), CD34 (vessels), CD20 (B-lymphocytes), CD3, CD4 and CD8 (T-lymphocytes), CD68 (macrophages), CD56 (NK-cells) and CD1a (dendritic cells) (all antibodies were fromVentana). Oestrogen receptors (ER) and progesterone receptors (PRs) were visualized using antibody 1D15 (Dako) and antibody NCL-PGR (Abbott Laboratories, Maidenhead, UK), according to a previously published protocol (34). The demonstration of oestrogen receptors (ER) were visualized using antibody 1D15 (Dako), and progesterone receptors (PRs), with antibody NCL-PGR (Abbott Laboratories, Maidenhead, UK), according to a previously published protocol [34]. The staining of ER and PRs was estimated using the "quick score" technique [34] as follows: slides were assessed for both the proportion of cells stained and staining intensity. Proportions were scored as 0 (no cells staining); 1) 1-25%; 2) 26-50%; 3) 50-75%; or 4) > 75% stained cells. The intensity was scored as 0, (no staining); 1, (weak); 2, (moderate); or 3, (strong staining). The two scores were added to give a final score of 0-7. A final score < 3 was regarded as negative.

### Digital video analysis

**Figure 1:**
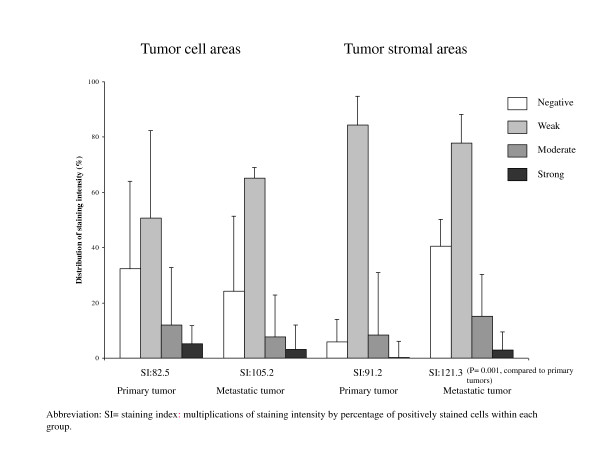
**Expression of COX-2 in primary and metastatic tumors**.

Microscopic images for quantitative analysis were recorded with a Leitz Aristoplane microscope equipped with a Leica DFC 320 digital camera. The Leica QWin Image Analysing system (Leica Microsystems Digital Imaging Solutions Ltd, Cambridge, UK) was used for morphometric analysis. Leica DFC320 is based on a 3.3 megapixel sensor. The staining intensity of COX-2, TGF-β, IL-10 and Ki67 in tumor cell areas and tumor stromal areas was quantified by measuring the colour value of red, green and blue colours (RGB), expressed in composite units. Density thresholds of RGB were set to quantify positive immunoreactivity of the red, green and blue colour components and these thresholds were fixed during the study. The number of pixels falling within each threshold (1 pixel = 0.172 μm) indicated the immunoreactivity reaction of each field and was recorded. The intensity of immunoreaction for each slide was expressed as the mean of RGB. Each slide was initially examined at 10× and 20× magnifications for an overall view. This practice allowed an area to be chosen as the most representative, with no tissue folding or overlapping, and minimal background staining. In each slide, ten different areas along a projected Z-line at 400× magnification comprising both tumor cell and stromal areas were systematically evaluated for the expression of COX-2, TGF-β, IL-10 and Ki67. Positive staining of COX-2 was assessed by the presence of marked diffuse brown cytoplasm in cancer cells (Figure 1). The staining intensity of COX-2 was scored as 0 (negative staining), 1 (weak), 2 (moderate), or 3 (strong staining) and the proportion of positive stained cells within each group were assessed. A combined staining index, SI, was calculated by multiplying the staining intensity (0, 1, 2, or 3) by the percentage of positively stained cells within each group (Figure 1) [34]. The expression of TGF-β, IL-10 and Ki67 was recorded in single non-epithelial cells within groups of epithelial cancer cells (tumor cell areas) and in the surrounding tumor stromalareas in central parts of the tumor (tumor stromal areas).

### Statistical analysis

Differences in staining intensity between primary tumors and metastases were analyzed By Wilcoxon signed rank test. Disease-specific survival was determined from the date of initial diagnosis to the time of breast cancer death. The risk of death from breast cancer in high (above median) and low (below median) staining intensity groups was compared by Kaplan-Meier survival analysis and log-rank test. COX-proportional hazards regression models were used to model the outcome death as a function of staining intensity. Age, histological grade (1-3), tumor size, oestrogen- and progesterone receptor positivity (yes/no), were included in the models in separate analyses to adjust for possible confounding. Her2-neu was done in few patients and was therefore excluded from these analyses. Pearson's product moment correlation coefficient and Spearman's rank correlation were used in the reproducibility analysis. A two-sided *p *value < 0.05 was considered statistically significant. The SPSS 16.0 software package was used in all analyses (SPSS Inc., Chicago, IL, USA). This study was approved by the Regional Committee for Research Ethics (REK, ref.200303108- 3/IAY/400), and this study was supported by grants from the Northern Norway regional Health Authority (Helse Nord RHF).

## Results

### Clinical features

Patients' characteristics are listed in Table 1. Mean age was 61.3 years (range, 39-89). Fifteen patients died during the follow-up, and mean survival time was 45.6 months (range, 36-120 months). Histological grade 2 was the most common (53%). Except for two, all patients had metastases to axillary lymph nodes at initial diagnose. Tumor size was > 10 mm in 36 (94.7%) patients. Mean tumor size at histological grade 1, 2 and 3 was 2, 4, and 3 mm, respectively. Oestrogen and progesterone hormone-receptor status were done in all tumors, 29 was oestrogen positive and 15 were progesterone positive.

**Table 1:** Patients demographics and clinical characteristics (N = 38)

	Histological differention grade		
	**1**	**2**	**3**

Mean age (range), years	52.1 (39-60)	62.3 (45-88)	63.8 (45-89)

	n = 6	n = 20	n = 12

Hormone receptor status			

ER+	5	19	5

ER-	1	1	8

PGR+	3	11	0

PGR-	3	9	6

Her2-Neu+	1	0	6

Her2-Neu-	2	4	2

Tumor size (mm)			

0 - 10	-	1	1

11 - 20	2	9	3

21 - 30	1	6	4

31 - 40	-	3	-

> 40	3	1	4

Mean	2	4	3

### Expression of COX-2, TGF-β, IL-10 and Ki67

**Figure 2:**
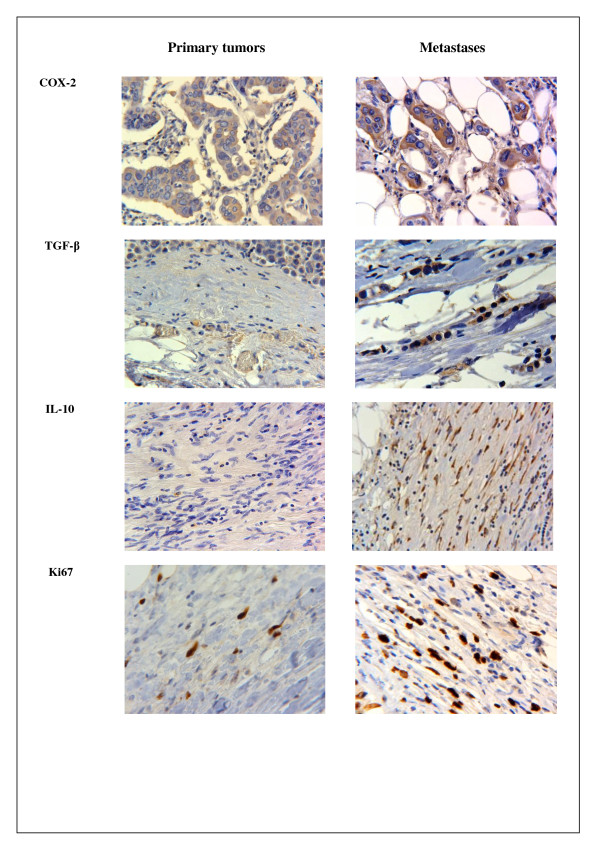
**Expression of COX-2, TGF-β, IL-10 and Ki67 in tumor cells and stromal cells areas of primary and metastases**.

Figures 1 and 2, and Table 2 illustrate the COX-2, TGF-β, IL-10 and Ki67 staining pattern in tumor cell areas and tumor stromal areas of the primary specimens and their corresponding lymph node metastases. The expression of COX-2, TGF-β, and IL-10 were predominantly cytoplasmic. The overall expression of COX-2 was higher in the metastases compared to the primary tumors (*p *< 0.001) (Figure 1). In primary tumors as well as in the metastases, the expression of COX-2 was highest in the tumor stromal areas (both *p *< 0.001). The staining indexes (SI) for COX-2 in tumor stromal areas were significantly higher in the metastases (121.3) compared to the primary tumors (91.2).

**Table 2:** Distribution of TGF-β, IL-10 and Ki67 in tumor cell areas and tumor stromal areas and their corresponding metastases.

		TGF-β	IL-10	Ki67
TP	Median (IQR)	760 (580-862)	395 (204-715)	676 (545-764)

SP	Median (IQR)	250 (119-377)	610 (291-791)	326 (238-454)

TM	Median (IQR)	781 (693-904)	656 (433-887)	419 (165-723)

SM	Median (IQR)	220 (92-273)	426 (255-273)	751 (539-855)

The values are pr 1000 counted cells.

Abbreviation; IQR: interquartile range; TP: tumor cell areas of primary tumors; SP: tumor stromal areas of primary tumors; TM: tumor cell areas of metastatic tumors; SM: stromal cell areas of metastatic tumors.

**Figure 3:**
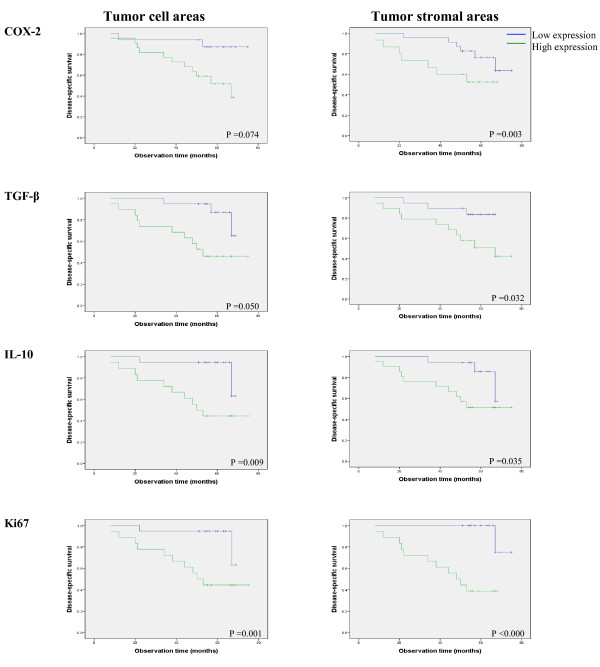
**Disease-specific survivals in tumor cell areas and tumor stromal areas of metastatic tumors**.

The expression of TGF-β was highest in the tumor cell areas of both primary tumors and metastases (both *p *< 0.001) (Table 2). IL-10 was expressed in cells with morphological features of macrophages and lymphocytes. The stromal expression was highest in the primary tumors (*p *< 0.001), whereas the tumor cell expression was highest in the metastases (*p *< 0.001) (Table 2). Ki67 expression (Table 2) was higher in stromal areas of the metastases (*p *< 0.001), whereas in primary tumors, the expression was highest in tumor cell areas (*p *< 0.001). Using the median as cut-off, the tumor and stromal expressions of the different markers Were categorized into a high versus low staining group. High expression of COX-2 was seen in tumor stromal areas of both primary tumors and the metastases (Figure 3 and Table 3, *p *= 0.020, *p *= 0.003, log rank test). High stromal staining intensity in the primary tumors was associated with a 3.9 (95% CI 1.1-14.2) times higher risk of death compared to the low staining group (*p *= 0.036) (Table 3). After adjustment for age, histological grade, oestrogen and progesterone receptor positivity the risk estimate was weakened, but remained borderline significant. The corresponding unadjusted and fully adjusted risk estimates for the stromal areas in the metastases were 6.8 (1.5-30.4) and 8.3 (4.2-27.7) (Table 3). High stromal expression of TGF-β in primary tumors wasassociated with increased mortality (HR 5.2, 95% CI 1.1-24.0, *P *= 0.035). High IL-10 expression in tumor cell areas (*p *= 0.018) and stromal areas (*p *= 0.003) in the primary tumors predicted mortality, whereas high staining intensity in stroma of the metastases was borderline associated (*p *= 0.057). Ki67 expression in tumor cell areas and stromal areas of the metastases was independently associated with breast cancer mortality.

**Table 3:** Risk of death from breast cancer in high staining intensity groups compared to low staining intensity groups

		Log Rank test	Unadjusted HR (95% CI)	Multivariate adjusted* HR (95% CI)
**COX-2**	TP	*P *= 0.322	0.6 (0.2-1.7), *P *= 0.339	0.6 (0.2-2.2), *P *= 0.485

	SP	*P *= 0.020	3.9 (1.1-14.2), *P *= 0.036	3.5 (0.8-14.7), *P *= 0.059

	TM	*P *= 0.074	2.3 (0.8-6.9), *P *= 0.121	1.5 (0.4-6.0), *P *= 0.592

	SM	*P *= 0.003	6.8 (1.5-30.4), *P *= 0.013	8.3 (4.2-27.7), *P *0.024

**TGF-β**	TP	*P *= 0.984	1.0 (0.3-2.8), *P *= 0.989	2.2 (0.6-8.8), *P *= 0.249

	SP	*P *= 0.839	1.1 (0.4-3.3), *P *= 0.842	5.2 (1.1-24.0), *P *= 0.035

	TM	*P *= 0.052	0.4 (0.1-4.1), *P *= 0.069	1.4 (0.3-6.6), *P *= 0.701

	SM	*P *= 0.032	3.0 (1.0-9.2), *P *= 0.046	2.3 (0.7-8.0), *P *= 0.174

**IL-10**	TP	*P *= 0.130	2.2 (0.8-6.4), *P *= 0.149	5.2 (1.3-20.6), *P *= 0.018

	SP	*P *= 0.384	1.7 (0.5-5.3), *P *= 0.400	1.1 (1.0-1.8), *P *= 0.003

	TM	*P *= 0.009	5.7 (1.3-25.6), *P *= 0.023	4.7 (0.8-25.5), *P *= 0.076

	SM	*P *= 0.035	4.4 (1.0-19.7), *P *= 0.053	4.0 (0.8-20.7), *P *= 0.057

**Ki67**	TP	*P *= 0.257	1.8 (0.6-5.3), *P *= 0.275	1.2 (0.3-4.7), *P *= 0.771

	SP	*P *= 0.369	1.7 (0.5-6.1), *P *= 0.387	1.1 (0.2-6.5), *P *= 0.959

	TM	*P *= 0.001	7.7 (1.7-35.2), *P *= 0.008	7.4 (1.4-33.3), *P *= 0.020

	SM	*P *< 0.000	3.6 (1.0-12.9), *P *= 0.031	1.1 (1.0-1.8), *P *= 0.002

*Adjusted for age, histology (grade 1, 2 and 3, tumor size, oestrogen- and progesterone receptor positivity (yes/no).

Abbreviations: TP: tumor cell areas of primary tumors; SP: tumor stromal areas of primary tumors; TM: tumor cell areas of metastases; SM: tumor stromal areas of metastases

## Discussion

In both primary tumors and metastases, we observed higher expression of COX-2 in the Tumor stromal areas than in the tumor cell areas. For IL-10, a higher stromal expression was seen in the primary tumors only, whereas for Ki67 the stromal expression was highest in the metastases. High stromal expression of COX-2 and Ki67 in metastases, as well as high stromal expression of TGF-β and IL-10 in primary tumors were independently associated to breast cancer mortality.

Mammary stromal tissue has a major role in the control and regulation of physiological processes in the breast. Likewise, during breast carcinoma development of the tumor stroma is believed to contribute in actively generating transformed lesions and tumors [35,36]. Evidence from genetic and clinical studies indicates that COX-2 up-regulation is one of the key steps in several preneoplastic lesions and cancers [37-41]. The role of COX-2 in the pre-invasive stages of breast tumorigenesis has been highlighted after recent publications, which linked the use of NSAIDs to decreased risk of breast cancer [42,43]. Our finding strengthens COX-2 as an important marker of breast cancer aggressiveness.

TGF-β signalling pathways are involved in many biological processes during embryogenesis, tissue homeostasis and mammary epithelial growth [44,45]. During transformation of a normal cell into a cancer cell, various components of the TGF-β signalling pathway may mutate, making the cell resistant to the effects of TGF-β [46,47]. TGF-β may suppress tumor growth in early stages, whereas at later stages TGF-β may enhance tumor growth [48]. In spite of a higher TGF-β expression in tumor cell areas, it was the stromal expression that was associated with breast cancer mortality in this study. This may indicate that stromal expression of TGF-β is particularly important in the early stage of tumor progression. Proliferative activity defined by Ki67 staining is associated with cancer progression and poor prognosis in a number of malignant tumors, including breast cancer [49]. Our study is in line with these reports, showing the highest expression of Ki67 in stroma of the metastases.

The production and action of COX-2, TGF-β and IL-10 are interrelated. Some experimental studies have shown that Th2 lymphocytes release high levels of IL-10 and thereby induce COX-2 expression [22]. IL-10 and TGF-β also cooperate to down-regulate immune responses. Studies on intestinal epithelial cells transgenic for IL-10 have shown that high TGF-β production also controls the ability to respond to TGF-β [21]. There are few studies comparing COX-2, TGF-β, and IL-10 expression in mammary tumor cell areas and tumor stromal areas. Except for TGF-β, the expression of COX-2, IL-10 and Ki67 in this study was higher in tumor stromal areas than in tumor cell areas. Our findings put emphasize on the surrounding stroma, supporting the hypothesis that the microenvironment surrounding tumor epithelium plays an important role in breast cancer progression. In future studies, both the tumor cell areas and the stromal areas should be investigated in primary breast cancer with or without metastases.

Most studies on human cancer tissues have evaluated immunohistochemical treated hotspot areas, where the staining intensity is highest. This is based on the assumption that one single section is representative for and reflects the histopathological pattern of the entire specimen. Moreover, the hotspot areas are evaluated without distinguishing between the tumor areas and the stromal areas. Instead of evaluating the protein expression in hotspot areas, we evaluated ten consecutively chosen fields along a projected Z-line in each tumor specimen. This approach was chosen to achieving a more representative picture of the tumor specimens and to put emphasizes on the role of stromal tissue in tumorigenesis.

In general, digital video analysis is regarded as being a more objective method with a higher sensitivity and reproducibility than light microscope and with better responsiveness to changes in cell counts [50,51]. However, there are technical pitfalls in this method, including background of the haematoxylin-eosin stained slides, the thickness of the slide, and tissue folding or overlapping, all which may cause bias in cell counting or assessment of protein expression. We excluded fields with tissue folding or overlapping from analysis. A shortcoming of this study is the small sample size. Our findings are on archived material, but should warrant a larger prospective analysis.

## Conclusions

In this study, immunohistochemical stromal expression of COX-2, TGF-β, IL-10 and Ki67 was associated to breast cancer mortality. Our findings heighten stroma as an active participant in the carcinogenesis of breast cancer.

## Competing interests

The authors declare that they have no competing interests.

## Authors' contributions

ER did the collection of tumor samples, and evaluated the immunohistochemical staining, the digital video analysis performed the statistical analysis and drafted the manuscript. RDU and ER performed the microscopic images for quantitative analysis. RDU performed the digital images. ER, LTB re-examined and histological graded the specimens. LTB participated in study design and extensively reviewed the manuscript. SHJ participated in data interpretation and reviewed the manuscript. All authors have read and approved the manuscript.
